# Comparative Evaluation of the Antioxidant and Immunomodulatory Activities of Carrot (*Daucus carota* L.) Aerial Parts and Roots Using Different Extraction Methods

**DOI:** 10.3390/foods14233993

**Published:** 2025-11-21

**Authors:** Sung-Sook Choi, Jae-Eun Lee, Hyo-Jun Lee, Kyung-Ae Lee

**Affiliations:** 1 Department of Food and Nutrition, Duksung Women’s University, Seoul 01370, Republic of Korea; choiss@duksung.ac.kr; 2 Graduate School of Biotechnology, College of Life Science, Kyunghee University, Yongin 17104, Republic of Korea; je8449@khu.ac.kr (J.-E.L.); gy9707@khu.ac.kr (H.-J.L.); 3 Department of Food and Nutrition, Anyang University, Anyang 14028, Republic of Korea

**Keywords:** *Daucus carota* L., aerial parts, antioxidant activity, immunomodulatory effect, by-products utilization

## Abstract

Carrot (*Daucus carota* L.) is a widely consumed root vegetable, yet its aerial parts, including leaves and stems, are typically discarded as agricultural by-products, despite their potential biological value. This study comparatively evaluated the antioxidant and immunomodulatory properties of carrot aerial and root parts extracted using hot water or 50% ethanol. Four extracts were prepared: aerial part hot-water (AP-W), aerial part ethanol (AP-E), underground part hot-water (UP-W), and underground part ethanol (UP-E). The total phenolic content (TPC, expressed as gallic acid equivalents; GAE) and total flavonoid content (TFC, expressed as quercetin equivalents; QE) were quantified using the Folin–Ciocalteu and aluminum nitrate colorimetric methods, respectively. Antioxidant capacities were determined by ABTS and DPPH radical scavenging assays, cytotoxicity was assessed in RAW 264.7 macrophages via the MTT assay, nitric oxide (NO) levels were measured using the Griess reaction, and cytokine (IL-6, TNF-α) concentrations were analyzed by ELISA. Among the extracts, AP-E exhibited the highest TPC (28.3 ± 0.3 µg GAE/mg extract) and TFC (18.2 ± 2.3 µg QE/mg extract), corresponding to the strongest ABTS (92.3 ± 2.5%) and DPPH (72.4 ± 7.3%) radical scavenging activities. None of the extracts demonstrated cytotoxicity below 400 µg/mL. Under basal conditions, AP-W and UP-W significantly enhanced NO production (9.5 ± 1.3 µM and 7.7 ± 1.2 µM, respectively), while co-treatment with LPS markedly reduced NO levels in AS-E (2.3 ± 0.2 µM). Consistently, AP-W and UP-W elevated cytokine secretion (IL-6: 3462.1 ± 349.7 pg/mL and 1749.4 ± 55.4 pg/mL; TNF-α: 15,245.2 ± 771.0 pg/mL and 14,719.1 ± 329.8 pg/mL), whereas AP-E (400 µg/mL) significantly suppressed IL-6 (3938.6 ± 268.7 pg/mL) and TNF-α (11,869.0 ± 721.1 pg/mL) under LPS-stimulated conditions. Collectively, these results indicate that hot-water extracts of carrot parts exert immunostimulatory activity, whereas ethanol extracts possess potent anti-inflammatory potential. The aerial parts of carrots, often regarded as waste biomass, exhibit comparable or superior bioactivities to the roots, underscoring their potential utility as promising functional food ingredients.

## 1. Introduction

Carrot (*Daucus carota* L.) is one of the most widely consumed root vegetables worldwide and is recognized for its high levels of carotenoids, phenolic compounds, and dietary fiber, which contribute to various antioxidant and health-beneficial effects [1,2]. While carrot roots and their processing residues have been utilized in food and industrial applications [3,4], the aerial parts, including leaves and stems, are typically discarded after harvest, resulting in the loss of potentially valuable biological resources and contributing to agricultural waste [5].

A growing number of studies have reported that by-products from root vegetables often contain substantial amounts of nutrients and bioactive compounds [5]. Carrot leaves, in particular, are known to possess high levels of phenolic acids and flavonoids with notable antioxidant and anti-inflammatory properties [6]. Nevertheless, most previous research has focused on isolated components—such as roots alone, leaves alone, or cultivar differences—rather than on a direct comparison of biological activities between the aerial and underground parts [6,7]. Therefore, a more integrated and systematic evaluation of the functional properties of different carrot parts is still needed.

In natural product research, solvent extraction plays a critical role in determining the yield and profile of bioactive compounds [8]. Among various extraction methods, hot-water and aqueous ethanol extractions are widely used in both laboratory and industrial applications owing to their safety, scalability, and efficiency [9,10]. Hot-water extraction is particularly effective for isolating highly polar bioactive constituents such as phenolics, flavonoids, and polysaccharides, which are responsible for antioxidant and immune-enhancing activities [11,12,13]. In contrast, extraction with aqueous ethanol facilitates the recovery of a wider spectrum of bioactive molecules, including moderately polar and semi-nonpolar compounds such as aglycone flavonoids, terpenoids, and certain lipid-soluble phenolics [8]. Due to these complementary characteristics, both extraction approaches have been extensively applied for the development of functional food and nutraceutical materials with enhanced antioxidant, anti-inflammatory, and immunomodulatory potential [11,14,15].

The immune system plays a crucial role in maintaining homeostasis and defending the host against pathogens [16]. In particular, macrophages play a pivotal role in immune signaling and contribute significantly to innate immunity [17]. Macrophages regulate immune responses by producing various mediators such as nitric oxide (NO) and cytokines, which play essential roles in host defense and immune regulation [18]. The immunoregulatory functions of macrophages are known to be influenced by various bioactive compounds [19]. Natural products, in particular, have attracted increasing attention because of their broad immunomodulatory potential, and numerous studies have explored their roles in enhancing immune regulation and suppressing inflammation [20]. Consequently, plant extracts and their derived bioactive compounds have been widely employed to evaluate their immune-related activities, demonstrating that various bioactive constituents can modulate immune responses through macrophage-mediated mechanisms [21,22].

Therefore, this study aimed to comparatively evaluate the antioxidant and immunomodulatory activities of carrot extracts obtained from underground (roots) and aerial (leaves and stems) parts to elucidate their biological differences. Furthermore, this work seeks to enhance the functional value and utilization of underutilized parts, thereby promoting their potential application as natural functional food materials.

## 2. Materials and Methods

### 2.1. Materials

Potassium persulfate, ABTS [2,2′-azino-bis(3-ethylbenzothiazoline-6-sulphonic acid)], DPPH [2,2′-diphenyl-1-picrylhydrazyl], ascorbic acid, sodium carbonate, 2 N Folin–Ciocalteu’s phenol reagent, gallic acid, and aluminum nitrate nonahydrate were purchased from Sigma-Aldrich (St. Louis, MO, USA). Ethanol (99.5%, Samchun Chemical Co., Ltd., Seoul, Republic of Korea) was used as an extraction solvent. Dulbecco’s Modified Eagle’s Medium (DMEM), fetal bovine serum (FBS), and antibiotic–antimycotic solution were obtained from GenDEPOT (Katy, TX, USA). Lipopolysaccharide (LPS), dimethyl sulfoxide (DMSO), the soluble form of MTT [3-(4,5-dimethylthiazol-2-yl)-2,5-diphenyltetrazolium bromide], and the Griess reagent were purchased from Sigma-Aldrich (St. Louis, MO, USA). Enzyme-linked immunosorbent assay (ELISA) kits for cytokine quantification were purchased from R&D Systems (Minneapolis, MN, USA). A microplate reader (FilterMax F5, Molecular Devices, San Francisco, CA, USA) was used to measure absorbance. The freeze-dryer (FD 8508, IlShin Biobase Co., Ltd., Dongducheon, Republic of Korea) was used for sample lyophilization.

### 2.2. Preparation of Extracts

The carrot (*Daucus carota* L.) samples used in this study were purchased from a local farm in Hongseong County, Chungcheongnam-do, Korea. The aerial (leaves and stems) and root parts were separated, washed thoroughly with distilled water, and dried in a hot-air dryer at 45 °C for 72 h. The dried samples were then ground into fine powders using a laboratory grinder and stored in airtight containers at room temperature until extraction. Dried powders of carrot aerial and underground parts were each extracted with hot water for 1 h and with 50% ethanol. The extracts were filtered through Whatman filter paper (Grade 2, GE Healthcare Life Sciences, Chicago, IL, USA), concentrated under reduced pressure, and freeze-dried (FD 8508; condenser temperature −80 °C; ice capacity 8 L; ultimate vacuum 5 mTorr) to obtain powdered extracts, which were stored for further experiments. The resulting extracts were designated as AP-W (aerial part, hot-water extract), AP-E (aerial part, 50% ethanol extract), UP-W (underground part, hot-water extract), and UP-E (underground part, 50% ethanol extract).

### 2.3. Total Phenolic Content (TPC)

Total phenolic content was measured using the Folin–Ciocalteu reagent. Each extract was mixed with 2 N Folin–Ciocalteu’s phenol reagent and 2% Na_2_CO_3_ solution, incubated in the dark for 30 min, and the absorbance was measured at 750 nm using a microplate reader (FilterMax F5; Molecular Devices, San Francisco, CA, USA). Gallic acid served as the calibration standard, and the data were expressed as micrograms of gallic acid equivalents per milligram of extract (µg GAE/mg extract).

### 2.4. Total Flavonoid Content (TFC)

Total flavonoid content was determined by the aluminum nitrate colorimetric method. The extract was sequentially mixed with 80% ethanol, 10% aluminum nitrate, and 1 M potassium acetate, incubated for 30 min in the dark, and the absorbance was measured at 415 nm. Quercetin was used as the reference standard. Data were presented as micrograms of quercetin equivalents per milligram of extract (µg QE/mg extract).

### 2.5. Radical Scavenging Activity (ABTS and DPPH Assays)

For the ABTS analysis, a mixture containing 7.4 mM ABTS and 2.6 mM potassium persulfate was freshly prepared and stored at 4 °C for 24 h in the dark to allow formation of the ABTS^+^ radical cation. After incubation, the solution was diluted with distilled water to obtain the working ABTS reagent. The extract was serially diluted to final concentrations of 1, 2, 4, and 8 mg/mL, mixed with the working solution, and incubated for 30 min in the dark at room temperature. The absorbance was then measured at 734 nm using a microplate reader.

For the DPPH assay, a 2 mM 2,2-diphenyl-1-picrylhydrazyl solution was prepared in 80% methanol and diluted to a suitable working concentration. The extract was adjusted to the same concentration range as used for the ABTS assay and reacted with the DPPH solution for 30 min at room temperature under light-protected conditions. The absorbance was then measured at 514 nm using the same reader.

ABTS and DPPH assays were employed to assess the extracts’ ability to scavenge free radicals. The percentage of inhibition was calculated using the following equation:

$$\mathrm{Inhibition}\;(\%)\;=\;\frac{\left[(A\_control-A\_blank)-(A\_sample-A\_sample\;blank)\right]}{\left(A\_control-A\_blank\right)}\times 100$$

where A_control is the absorbance of the radical solution (ABTS or DPPH) without the sample, A_blank is the absorbance of the solvent without ABTS/DPPH and sample, A_sample is the absorbance of the reaction mixture containing the sample and radical solution, and A_sample blank is the absorbance of the sample solution without ABTS/DPPH.

### 2.6. Cell Culture and Cytotoxicity

RAW 264.7 cells obtained from the Korean Cell Line Bank (Seoul National University, Seoul, Republic of Korea) were cultured in Dulbecco’s Modified Eagle’s Medium (DMEM) containing 10% fetal bovine serum (FBS) and 1% antibiotic–antimycotic solution at 37 °C in a humidified incubator with 5% CO_2_. When cell confluence reached approximately 90%, the cells were seeded into 96-well plates at a density of 2 × 10^4^ cells/well. For the immunostimulatory experiment, the cells were treated with four carrot extracts (AP-W, AP-E, UP-W, and UP-E) at dose levels of 100, 200, and 400 μg/mL for 24 h. For the anti-inflammatory experiment, inflammation was induced by co-treatment with lipopolysaccharide (LPS, 1 µg/mL) and each extract at dose levels of 100, 200, and 400 µg/mL for 24 h; an LPS-treated group served as the positive control. After the treatments, MTT reagent (0.5 mg/mL) was added to the wells and allowed to react for 3 h. The formed formazan was then dissolved in dimethyl sulfoxide (DMSO), and its absorbance was quantified at 570 nm using a microplate reader. The MTT assay was conducted to evaluate the cytotoxicity of each extract and to confirm that the observed changes in NO and cytokine production were not due to reduced cell viability. The concentrations of 100, 200, and 400 μg/mL were determined based on previous studies that investigated the biological activities of carrot and various plant extracts using comparable concentration ranges [23,24].

### 2.7. Nitric Oxide (NO) Assay

RAW 264.7 cells were seeded into 96-well plates at a density of 2 × 10^4^ cells/well. For the immunostimulatory experiment, cells were treated with carrot extracts (AP-W, AP-E, UP-W, and UP-E) at dose levels of 100, 200, and 400 μg/mL for 24 h. For the anti-inflammatory experiment, inflammation was induced by co-treatment with lipopolysaccharide (LPS, 1 μg/mL) and each extract at the same dose levels (100, 200, and 400 μg/mL). An LPS-treated group served as the positive control. After incubation, the culture supernatants were collected and combined with an equal amount of Griess reagent, after which the absorbance was determined at 570 nm using a microplate reader.

### 2.8. Enzyme-Linked Immunosorbent Assay (ELISA)

RAW 264.7 cells were cultured and treated with carrot extracts (AP-W, AP-E, UP-W, and UP-E) at concentrations of 100, 200, and 400 μg/mL, with or without lipopolysaccharide (LPS, 1 μg/mL) for 24 h. The supernatants were collected to assess the effects of the extracts on cytokine production in macrophages. The levels of IL-6 and TNF-α were quantified using ELISA kits (R&D Systems, according to the manufacturer’s instructions).

### 2.9. Statistical Analysis

All data are reported as the mean ± standard deviation (SD) from three independent experiments. Statistical evaluations were carried out using SPSS Statistics 29 (IBM Corp., Armonk, NY, USA). Group comparisons were conducted through one-way ANOVA followed by Tukey’s post hoc test, and pairwise comparisons between the control and each treatment group (in the immunostimulatory assay) or between the LPS-treated and LPS + extract-treated groups (in the anti-inflammatory assay) were evaluated using an independent samples *t*-test. Statistical significance was considered at *p* < 0.05.

## 3. Results

### 3.1. Total Phenolic and Flavonoid Contents of Carrot Extracts

**Figure 1:**
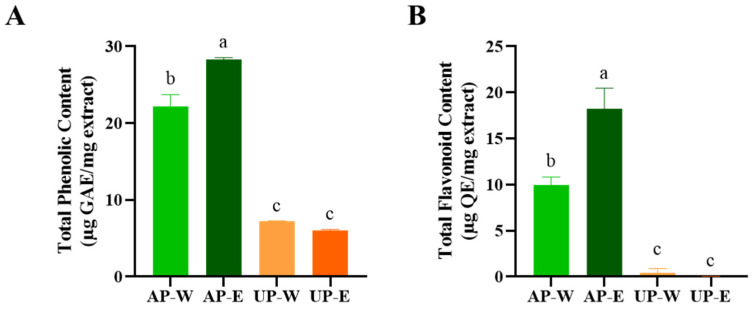
Comparison of phenolic and flavonoid levels in carrot extracts. (**A**) Total phenolic content (TPC) and (**B**) total flavonoid content (TFC) of carrot aerial and underground part extracts. Values are shown as mean ± SD (*n* = 3). Distinct superscript letters (a–c) above each bar indicate statistically significant group differences as determined by Tukey’s test (*p* < 0.05). AP-W, aerial part hot-water extract; AP-E, aerial part 50% ethanol extract; UP-W, underground part hot-water extract; UP-E, underground part 50% ethanol extract.

The total phenolic and flavonoid contents are key indicators of antioxidant capacity, as these compounds contribute to radical scavenging [25]. The total phenolic and flavonoid contents of carrot extracts were evaluated to compare the distribution of bioactive compounds between the aerial and underground parts (Figure 1). Both total phenolic content (TPC) and total flavonoid content (TFC) were markedly higher in the aerial part extracts than in the underground ones. Among the four extracts, the 50% ethanol extract of the aerial part (AP-E) exhibited the highest levels, with TPC of 28.3 ± 0.3 µg GAE/mg extract and TFC of 18.2 ± 2.3 µg QE/mg extract, followed by the aerial hot-water extract (AP-W). In contrast, both underground extracts (UP-W and UP-E) showed comparatively lower phenolic and flavonoid contents. These findings indicate that the aerial parts of carrots, particularly those extracted with ethanol, are richer in polyphenolic and flavonoid compounds, which may contribute to their stronger antioxidant potential observed in subsequent analyses.

### 3.2. Antioxidant Effect of Carrot Extracts

**Figure 2:**
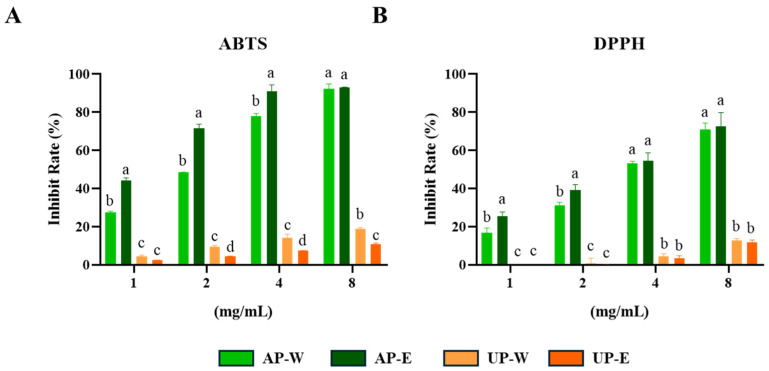
ABTS and DPPH free-radical quenching activities of carrot extracts. (**A**) ABTS and (**B**) DPPH radical scavenging activities of carrot aerial and underground part extracts. Values are expressed as mean ± SD (*n* = 3). Different letters (a–d) above the bars indicate significant differences among groups according to Tukey’s test (*p* < 0.05).

Both ABTS and DPPH assays are widely used to assess the free-radical–scavenging capacity of plant extracts, reflecting their overall antioxidant potential [26]. The antioxidant capacities of carrot aerial and underground part extracts were evaluated through ABTS and DPPH free-radical assays (Figure 2). Both assays showed that the aerial part extracts possessed significantly stronger radical scavenging activity than the underground part extracts. At the highest tested concentration (8 mg/mL), the 50% ethanol extract of the aerial part (AP-E) showed the strongest activity, with 92.3 ± 2.5% inhibition in the ABTS assay and 72.4 ± 7.3% scavenging in the DPPH assay, followed by the aerial hot-water extract (AP-W). In contrast, both underground extracts (UP-W and UP-E) displayed considerably lower radical scavenging activities. These findings suggest that the superior antioxidant properties of the aerial extracts may be attributed to their higher phenolic and flavonoid levels, consistent with the TPC and TFC results. Similar trends have been reported in previous studies, where carrot leaves exhibited stronger antioxidant capacity than roots owing to their abundant phenolic constituents [27,28].

### 3.3. Cytotoxicity of Carrot Extracts in RAW 264.7 Cells

**Figure 3:**
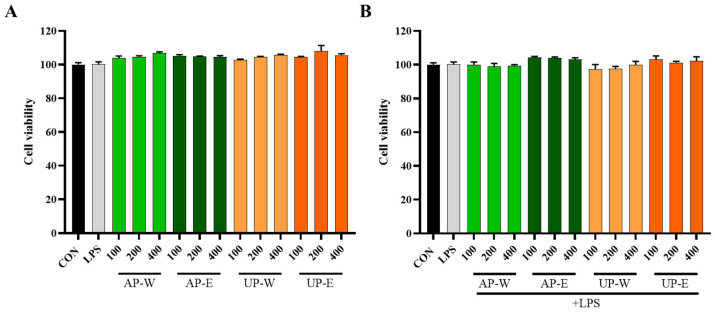
Cell viability of RAW 264.7 macrophages treated with carrot extracts. (**A**) RAW 264.7 macrophages treated without LPS and (**B**) treated with LPS were exposed to carrot aerial and underground part extracts for 24 h to evaluate cytotoxicity. Data are presented as the mean ± SD (*n* = 3). Statistical analysis was performed using independent *t*-tests compared with the control group. No significant decreases were observed in any treatments (*p* > 0.05), indicating the absence of cytotoxicity.

RAW 264.7 macrophage survival following treatment with carrot extracts was examined via the MTT assay (Figure 3). All extracts (AS-W, AS-E, UG-W, and UG-E) showed no cytotoxicity at concentrations up to 400 μg/mL, and cell viability remained above 95%. Moreover, co-treatment with lipopolysaccharide (LPS, 1 μg/mL) did not affect cell viability, indicating that neither the extracts nor their combination with LPS exerted any cytotoxic effects on macrophages.

### 3.4. Modulation of Carrot Extracts on Nitric Oxide Generation in RAW 264.7 Cells

**Figure 4:**
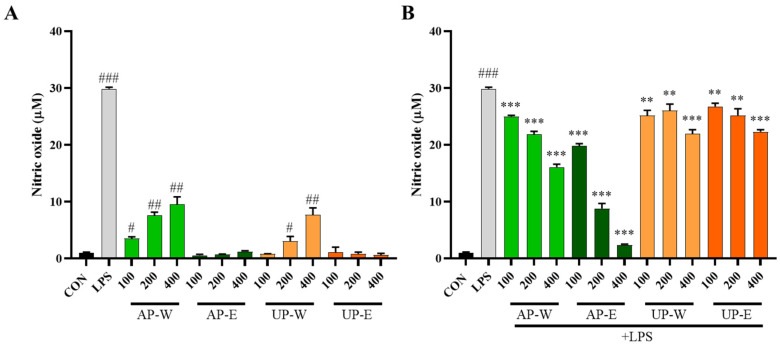
Nitric oxide production in RAW 264.7 macrophages treated with carrot extracts under non-stimulated and LPS-stimulated conditions. (**A**) Non-stimulated and (**B**) LPS-stimulated RAW 264.7 macrophages were treated with carrot aerial and underground part extracts for 24 h to evaluate nitric oxide (NO) production. Data are presented as mean ± SD (*n* = 3). Statistical differences versus the control group are denoted by # *p* < 0.05, ## *p* < 0.01, ### *p* < 0.001, while comparisons with the LPS-treated group are marked with ** *p* < 0.01, *** *p* < 0.001.

Nitric oxide is a major signaling molecule produced by macrophages during immune activation [29]. Nitric oxide (NO) production was measured to evaluate the immunostimulatory and anti-inflammatory effects of carrot extracts (Figure 4). Under non-stimulated conditions (without LPS), the hot-water extracts (AP-W and UP-W) markedly enhanced NO production compared with the control group, with AP-W and UP-W producing 9.5 ± 1.3 µM and 7.7 ± 1.2 µM of NO, respectively, indicating immune-stimulatory activity. In contrast, the ethanol extracts (AP-E and UP-E) showed minimal effects, suggesting that water-soluble components such as polysaccharides may be responsible for macrophage activation. Under LPS-stimulated conditions, NO production was substantially increased by LPS treatment; however, co-treatment with the aerial part extracts significantly reduced NO production in a dose-responsive pattern. In particular, the 50% ethanol extract of the aerial part (AP-E) exhibited the strongest inhibitory effect, lowering NO production to 2.3 ± 0.2 µM, followed by the hot-water extract (AP-W), whereas both underground part extracts (UP-W and UP-E) showed weak suppression. These results indicate that the aerial part, especially the ethanol extract rich in phenolic compounds, possesses potent anti-inflammatory activity through inhibition of NO production.

### 3.5. Effects of Carrot Extracts on IL-6 and TNF-α Secretion in RAW 264.7 Cells

**Figure 5:**
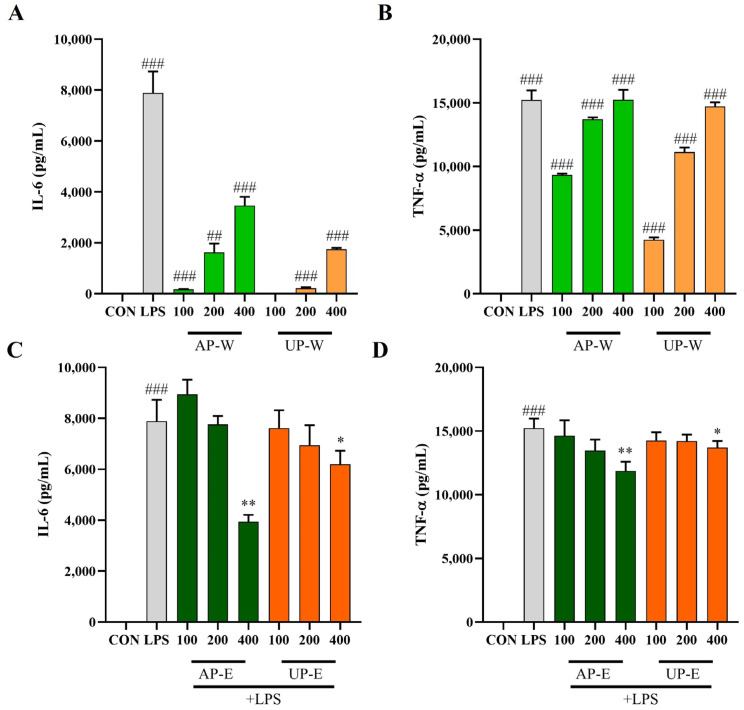
Effects of carrot extracts on IL-6 and TNF-α production in RAW 264.7 macrophages. (**A**,**B**) IL-6 and TNF-α levels in RAW 264.7 macrophages treated with hot-water extracts (AP-W and UP-W) under non-stimulated conditions. (**C**,**D**) IL-6 and TNF-α levels in cells treated with 50% ethanol extracts (AP-E and UP-E) under LPS-stimulated conditions. Data are presented as mean ± SD (*n* = 3). Statistical differences versus the control group are denoted by ## *p* < 0.01, ### *p* < 0.001, while comparisons with the LPS-treated group are marked with * *p* < 0.05, ** *p* < 0.01.

IL-6 and TNF-α constitute pro-inflammatory cytokines released by activated macrophages, serving as biomarkers for immune and inflammatory modulation [30]. To further investigate the immunomodulatory effects of carrot extracts, the levels of IL-6 and TNF-α were measured in RAW 264.7 macrophages under non-stimulated and LPS-stimulated conditions (Figure 5). Under non-stimulated conditions (without LPS), treatment with the hot-water extracts significantly increased cytokine secretion. AP-W elevated IL-6 to 3462.1 ± 349.7 pg/mL and TNF-α to 15,245.2 ± 771.0 pg/mL, while UP-W produced 1749.4 ± 55.4 pg/mL of IL-6 and 14,719.1 ± 329.8 pg/mL of TNF-α, indicating macrophage activation and immune-enhancing effects. Under LPS-stimulated conditions, cytokine levels were markedly elevated by LPS treatment, whereas co-treatment with the ethanol extracts (AP-E and UP-E) notably suppressed IL-6 and TNF-α production in a concentration-dependent manner. In particular, AP-E (400 µg/mL) reduced IL-6 to 3938.6 ± 268.7 pg/mL and TNF-α to 11,869.0 ± 721.1 pg/mL, showing the most pronounced inhibitory effect. These results suggest that phenolic-rich compounds in the aerial ethanol extract exert anti-inflammatory activity by downregulating pro-inflammatory cytokine expression.

## 4. Discussion

This study revealed that the aerial portions of carrots contain greater amounts of bioactive compounds and exhibit stronger antioxidant and immunomodulatory properties than the root parts. While several previous reports have described and quantified phenolic constituents from either carrot leaves or roots [31,32,33], comparative evaluations of both tissues under identical analytical conditions remain scarce. Hence, the present findings provide new insight into how compositional variation among plant parts is closely linked to differences in antioxidant potential.

The elevated total phenolic and flavonoid levels detected in the aerial extracts may result from physiological and environmental influences. Because photosynthetic tissues are continuously exposed to light and oxidative challenges, they tend to activate the biosynthetic machinery of secondary metabolites such as polyphenols and flavonoids as part of their protective response [34,35]. Previous investigations have also demonstrated that light exposure modulates the production of diverse classes of secondary metabolites, including phenolics, flavonoids, anthocyanins, terpenoids, and alkaloids, by enhancing the transcription and activity of key biosynthetic enzymes [36,37]. Consequently, the greater accumulation of phenolic and flavonoid compounds in the aerial tissues may, at least in part, be associated with light-dependent metabolic regulation occurring in photosynthetically active organs.

At the molecular level, light perception in plants occurs through specific photoreceptors such as phytochromes (red/far-red light receptors), cryptochromes, and phototropins (blue-light receptors), which activate downstream signaling cascades that regulate secondary metabolism [37]. Upon light stimulation, these photoreceptors trigger the expression of transcription factors, including ELONGATED HYPOCOTYL 5 (HY5) and MYB family proteins, which directly control the transcription of key structural genes in the phenylpropanoid biosynthetic pathway—namely phenylalanine ammonia-lyase (PAL), chalcone synthase (CHS), and flavonol synthase (FLS) [38,39,40]. These enzymes are central to the biosynthesis of phenolic and flavonoid compounds and are upregulated in light-exposed tissues as part of the plant’s adaptive defense mechanism against photooxidative stress [38,40].

Nevertheless, as this work primarily assessed total phenolic and flavonoid contents rather than identifying individual compounds, additional phytochemical characterization is warranted to delineate and compare the specific constituents present in each extract. Such studies would help clarify the molecular mechanisms underlying the functional disparities observed between carrot aerial and root tissues.

In macrophages, the carrot extracts exhibited distinct biological effects depending on the extraction solvent and plant part. The hot-water extracts (AP-W and UP-W) showed immune-stimulatory activity under non-stimulated conditions, indicating their potential to activate macrophages. These results are consistent with the general activation patterns observed in previous immunostimulatory studies [41,42], suggesting that carrot extracts possess potential as immunomodulatory materials. These effects may be associated with the presence of water-soluble polysaccharides in the extracts. Numerous studies have reported that polysaccharides isolated from plant-derived hot-water extracts exhibit immune-enhancing properties in macrophages and immunosuppressed mouse models [43,44]. Such polysaccharides are known to promote nitric oxide production and cytokine secretion, thereby activating innate immune responses, which is in agreement with the present findings. Therefore, the immunostimulatory activity of carrot hot-water extracts is likely, at least in part, mediated by polysaccharide-rich fractions that enhance macrophage function. Further studies are required to isolate and structurally characterize these polysaccharides and to confirm their immunomodulatory effects through in vivo experiments.

Under LPS-stimulated conditions, the carrot extracts exhibited distinct anti-inflammatory activities depending on the extraction solvent and plant part. The aerial ethanol extract (AS-E) showed the most pronounced inhibitory tendency on nitric oxide production, which aligns with previous findings that ethanol extracts of carrot leaves exhibit greater anti-inflammatory potential than hot-water extracts [6]. Consistent with this trend, the ethanol extract of the aerial part also more effectively suppressed IL-6 and TNF-α production compared with that of the underground part. Collectively, these results suggest that the ethanol extract of carrot aerial parts has significant potential as a natural anti-inflammatory material.

Overall, this study highlights the functional potential of carrot aerial parts as valuable bioresources that are often discarded after harvest. The aerial extracts demonstrated strong antioxidant, anti-inflammatory, and immunostimulatory properties, depending on the extraction solvent. These findings suggest that compositional differences between the aerial and underground parts of carrots contribute to their distinct biological activities. The remarkable bioactivity of the aerial extracts, particularly the ethanol extract, indicates that the underutilized aerial portions of carrots can serve as promising sources of natural functional ingredients with both antioxidant and immune-regulating capacities. Further in vivo and phytochemical studies are warranted to identify the specific bioactive compounds responsible for these effects and to elucidate their underlying molecular mechanisms.

## 5. Conclusions

This study comparatively evaluated the antioxidant and immunomodulatory activities of carrot aerial and underground parts extracted with hot water and 50% ethanol. The results revealed that the aerial parts, particularly the 50% ethanol extract (AP-E), contained higher levels of total phenolic and flavonoid compounds and exhibited the strongest radical scavenging capacity. Moreover, the hot-water extracts (AP-W and UP-W) enhanced nitric oxide and cytokine (IL-6 and TNF-α) production in macrophages under non-stimulated conditions, indicating immune-stimulatory effects. In contrast, under LPS-stimulated conditions, the aerial ethanol extract (AP-E) effectively suppressed nitric oxide and pro-inflammatory cytokine production, demonstrating potent anti-inflammatory activity. Collectively, these findings suggest that carrot aerial parts, which are typically discarded as agricultural by-products, represent a valuable source of natural bioactive materials with antioxidant, immune-enhancing, and anti-inflammatory potential. Further in vivo studies and detailed structural characterization of the active compounds, including polysaccharides and phenolic constituents, are warranted to validate their physiological effects and practical applications as functional food ingredients.

## Data Availability

The original contributions of this study are included in the article. Further inquiries can be directed to the corresponding author.

## Abbreviations

The following abbreviations are used in this manuscript:

ABTS	2,2′-Azino-bis(3-ethylbenzothiazoline-6-sulfonic acid)
AP-E	Aerial part 50% ethanol extract
AP-W	Aerial part hot-water extract
CHS	Chalcone synthase
DPPH	2,2′-Diphenyl-1-picrylhydrazyl
FLS	Flavonol synthase
HY5	ELONGATED HYPOCOTYL 5
IL-6	Interleukin-6
LPS	Lipopolysaccharide
NO	Nitric oxide
PAL	Phenylalanine ammonia-lyase
TPC	Total phenolic content
TFC	Total flavonoid content
TNF-α	Tumor necrosis factor-alpha
UP-E	Underground part 50% ethanol extract
UP-W	Underground part hot-water extract
